# Early markers are present in both embryogenesis pathways from microspores and immature zygotic embryos in cork oak, *Quercus suber* L

**DOI:** 10.1186/s12870-014-0224-4

**Published:** 2014-08-21

**Authors:** Héctor Rodríguez-Sanz, José-Antonio Manzanera, María-Teresa Solís, Aránzazu Gómez-Garay, Beatriz Pintos, María C Risueño, Pilar S Testillano

**Affiliations:** Pollen Biotechnology of Crop Plants group, Biological Research Center, CIB-CSIC, Ramiro de Maeztu 9, 28040 Madrid, Spain; ETSI Montes, Technical University of Madrid, UPM, Ciudad Universitaria, 28040 Madrid, Spain; Department of Plant Physiology, Faculty of Biology, Complutense University of Madrid, UCM, Ciudad Universitaria, 28040 Madrid, Spain

**Keywords:** Somatic embryogenesis, Microspore embryogenesis, Anther culture, DNA methylation, 5-methyl-deoxy-cytidine, Pectin esterification, Cell wall, Auxin, IAA

## Abstract

**Background:**

In *Quercus suber*, cork oak, a Mediterranean forest tree of economic and social interest, rapid production of isogenic lines and clonal propagation of elite genotypes have been achieved by developing *in vitro* embryogenesis from microspores and zygotic embryos respectively. Despite its high potential in tree breeding strategies, due to their recalcitrancy, the efficiency of embryogenesis *in vitro* systems in many woody species is still very low since factors responsible for embryogenesis initiation and embryo development are still largely unknown. The search for molecular and cellular markers during early stages of *in vitro* embryogenesis constitutes an important goal to distinguish, after induction, responsive from non-responsive cells, and to elucidate the mechanisms involved in embryogenesis initiation for their efficient manipulation. In this work, we have performed a comparative analysis of two embryogenesis pathways derived from microspores and immature zygotic embryos in cork oak in order to characterize early markers of reprogrammed cells in both pathways. Rearrangements of the cell structural organization, changes in epigenetic marks, cell wall polymers modifications and endogenous auxin changes were analyzed at early embryogenesis stages of the two *in vitro* systems by a multidisciplinary approach.

**Results:**

Results showed that early embryo cells exhibited defined changes of cell components which were similar in both embryogenesis *in vitro* systems, cellular features that were not found in non-embryogenic cells. DNA methylation level and nuclear pattern, proportion of esterified pectins in cell walls, and endogenous auxin levels were different in embryo cells in comparison with microspores and immature zygotic embryo cells from which embryos originated, constituting early embryogenesis markers.

**Conclusions:**

These findings suggest that DNA hypomethylation, cell wall remodeling by pectin esterification and auxin increase are involved in early *in vitro* embryogenesis in woody species, providing new evidences of the developmental pattern similarity between both embryogenesis pathways, from microspores and immature zygotic embryos, in woody species.

## Background

*In vitro* embryogenesis systems constitute important tools for investigating the regulating mechanisms of embryo formation, as well as for biotechnological applications in plant breeding, propagation and conservation strategies. Two different embryogenesis pathways, from microspores and immature zygotic embryos, have been developed in many species for rapid production of isogenic lines and clonal propagation of elite genotypes respectively. Tree breeding strategies have focused on ways to reduce cycle time and improve the efficiency of selection; here, propagation of selected trees by somatic embryogenesis and genetic engineering approaches applied to haploids and double-haploid plants produced in short-times by *in vitro* microspore embryogenesis have a high potential. Nevertheless, due to their recalcitrance, the efficiency of embryogenesis *in vitro* systems in many woody species is still very low.

*Quercus* trees, oaks, are woody species of relevant economic and ecological interest; among them the cork oak, *Q. suber*, is a species of high social and economic relevance in the Mediterranean area as the natural source of cork. Different *in vitro* embryogenesis systems have been developed for *Quercus spps*., with variable efficiency [1-3]. Despite the work performed in the last decades, factors responsible for embryogenesis initiation and embryo development in established cultures are still largely unknown.

The search for molecular and cellular markers during early stages of *in vitro* embryogenesis constitutes an important goal in the identification of cells committed to the embryogenesis developmental program as opposed to those cells which are non-responsive to the embryogenic pathway, as well as in the elucidation of the cellular mechanisms underlying *in vitro* embryo progression. Changes in various cell activities and in the structural organization of subcellular compartments have been reported as accompanying the microspore reprogramming process in some herbaceous and woody species [4-11]. Increasing evidences have indicated the relevance of some cell features like epigenetic marks [12-15], cell wall components [10,16-19] and hormones [9] in the progression of *in vitro* organogenesis and embryogenesis in other systems, but no reports about the dynamics of these three cellular markers during *in vitro* early embryogenesis are available in two somatic embryogenesis pathways of the same species.

Dynamic changes between chromatin states are relevant in the transcriptional regulation during microspore development and reprogramming to embryogenesis [6] and epigenetic mechanisms play an essential role in the process of cellular differentiation allowing cells to be reprogrammed in order to generate new differentiation pathways [12]. DNA methylation constitutes a prominent epigenetic modification of the chromatin fiber, which becomes locked in a transcriptionally inactive conformation, thus leading to gene silencing. Stress-induced plant cell reprogramming involves changes in global genome organization, being the epigenetic modifications key factors of genome flexibility [13]. Previous studies have shown changes in DNA methylation levels and distribution patterns during microspore embryogenesis of *Brassica napus* and *Hordeum vulgare* [14,15], suggesting the existence of an epigenetic reprogramming after microspore induction to embryogenesis, but no information is available on DNA methylation dynamics during *in vitro* embryogenesis in trees.

Many of the molecular markers of *in vitro* embryogenesis and organogenesis have been found in cell walls [10,16-19]. Pectins are the major matrix components of dicotyledonous cell walls. Pectins are polymerized and methyl-esterified in the Golgi, and secreted into the wall as highly methyl-esterified forms. Subsequently, they can be modified by pectin methylesterases, which catalyse the demethylesterification of homogalacturonans domains of pectins. The relationship between the esterified and the non-esterified pectins, and their distribution in the plant cell walls is the result of different processes [20-22] and their proportion and dynamics are involved in many plant developmental processes. Changes in the distribution of pectins have been reported in young embryos generated from microspores of *Quercus suber* L. [8], *Citrus clementina* [23] and *Olea europaea* L. [10]. Abundant esterified pectins has been reported in the newly-formed walls of proliferating cells in different plant systems, like young microspore-derived embryos and root tip meristematic cells of herbaceous species [17,18], suggesting that a high proportion of esterified pectins in walls is a marker for proliferation events while abundant low-esterified pectins correspond to low cell proliferation rates [17,18].

The plant growth regulator (PGR) auxin, the predominant form of which is indole-3-acetic acid (IAA), is a major coordinating signal in the regulation of plant development, with key functions in many processes including embryo formation [24,25]. Despite it is widely known the stimulating effects of the addition of exogenous PGRs, including synthetic auxins like 2,4-D, on *in vitro* embryogenesis induction [9,26], little is known about endogenous levels of these regulators at the initial stages of embryogenesis. In cork oak somatic embryos, the quantification of IAA showed the highest concentration during early embryo development and decreased afterwards [27]. Recent work has reported preliminary results showing that endogenous IAA localized in cells of early multicellular embryos developed in isolated microspore cultures of *Brassica napus* [28].

Previous reports have indicated that microspore embryogenesis mimics zygotic embryogenesis in several species [7,9,14,29]. Proteomic analyses have approached the identification of protein expression patterns in *Q. suber* embryos originated by different *in vitro* systems during advanced developmental stages and maturation [30,31]. The phytohormones can act as signaling molecules for remodeling cell walls during cell elongation, as well as for modifying chromatin domains to activate gene expression programs. In this sense, the information about the dynamics of epigenetic marks, pectin esterification changes in walls and auxin during early embryogenesis could inform on possible correlations among these processes, giving new insights into the complex regulatory mechanisms controlling cell reprogramming and embryogenesis induction.

In the present work, we have performed a comparative analysis of *in vitro* embryogenesis of microspores and immature zygotic embryos in cork oak in order to characterize early markers of embryo cells, in contrast with non-responsive cells, in both pathways and to gain new insights into the cellular mechanisms involved in the *in vitro* embryogenesis induction in woody species, in which there is scarce information, as well as to provide new evidences of the developmental pattern similarity between both embryogenesis pathways, from microspores and zygotic embryos. We have analyzed the changes in DNA methylation, esterification of pectins in cell walls and endogenous auxin distribution, during early embryogenesis of the two *in vitro* systems.

The results presented here revealed differential features in early multicellular embryo cells constituting early embryogenic markers which are similar in both pathways originated from microspores and zygotic embryos, providing additional evidences to the developmental pattern analogy of embryogenesis pathways.

## Results

### Characterization of early developmental stages of embryogenesis of both microspore and zygotic embryo origins in *Quercus suber*

Two different embryogenic *in vitro* systems have been compared in cork oak, embryogenesis of microspores (through anther culture) and immature zygotic embryos, to analyze the presence and distribution patterns of various cellular components at early embryogenesis stages and to identify common markers present in the two embryogenic pathways at the initial stages.

**Figure 1:**
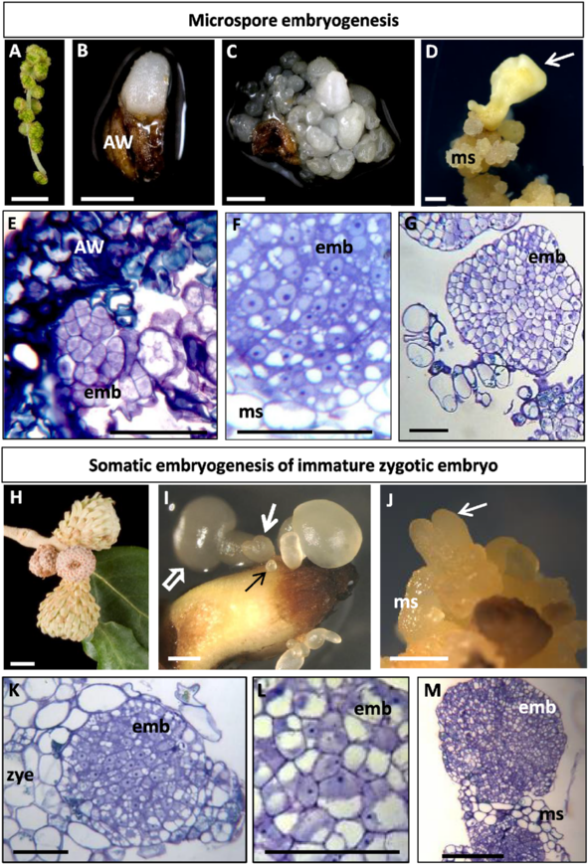
**Main developmental stages of** ***in vitro*** **embryogenesis in cork oak. A**-**G**: Microspore embryogenesis. **H**-**M**: Somatic embryogenesis of immature zygotic embryos. **A-D**, **H-J**: Explants and *in vitro* embryo development. **E-G**, **K-M**: Different stages of embryo development, resin sections stained by toluidine blue. **A**: Catkin selected for *in vitro* microspore embryogenesis. **B**, **C**: Multicellular and globular embryos (white structures) of different sizes emerging from inside anthers (AW, anther wall) after 20–30 days in culture. **D**: Secondary embryo (white arrow) emerging from embryogenic masses (ms). **E**: Early multicellular embryos (emb) formed inside the anther and surrounded by the tissue of the anther wall (AW). **F**: Cells of an early multicellular embryo at higher magnification. **G**: Large multicellular embryo formed by secondary embryogenesis attached to some cells of an embryogenic mass (ms). **H**: Acorn selected for *in vitro* somatic embryogenesis of immature zygotic embryos. **I**: Embryos of different developmental stages and sizes emerging from the immature zygotic embryo explant after 30 days in culture, early multicellular embryo (black thin arrow), globular, heart (white thick arrow) and cotyledonary (white open arrow) embryos. **J**: Secondary embryo (white arrow) at the torpedo stage originated from embryogenic masses (ms). **K**: Early multicellular embryo (emb) formed at the surface of an immature zygotic embryo (zye). **L**: Cells of an early multicellular embryo at higher magnification. **M**: Early multicellular embryos (emb) formed by secondary embryogenesis and emerging from inside an embryogenic mass (ms). Bars: **A, H**: 100 μm; **B, C, D, I, J**: 1 mm; **E, F, GK, L**: 50 μm; **M**: 200 μm.

Microspore embryogenesis was induced by heat treatment in anthers excised from catkins (Figure 1A) at the developmental stage in which they contained microspores at the responsive stage of vacuolated microspores. After 20–25 days in culture, white small multicellular and globular embryos emerged from inside the anther (Figure 1B, C); these embryos further developed to heart, torpedo and cotyledonary embryos. Mature cotyledonary embryos produced by microspore embryogenesis were subcultured monthly on medium without growth regulators producing embryogenic masses (“ms” in Figure 1D) from which new embryos emerged by secondary embryogenesis (arrow in Figure 1D).

Microscopic analysis of culture samples at early developmental stages showed small multicellular embryos (“emb” in Figure 1E) of different sizes inside the anthers and emerging out of the them. The early multicellular embryos originated by either direct or secondary embryogenesis showed a typical structural organization consisting in a more or less rounded structure formed by small cells, with characteristic features of active proliferating cells. The cells of these so-called early multicellular embryos were small, with medium-large nucleus, slightly dense cytoplasm and low vacuolation (“emb” in Figure 1E, F, G), very different from the cells of the anther wall tissues (“AW” in Figure 1E) and embryogenic masses (“ms” in Figure 1F, G) which were of larger size and highly vacuolated.

Immature pollinated acorns (Figure 1H) were the source of zygotic embryos which were cultured and used for somatic embryogenesis induction. Around one month after the culture initiation, early multicellular embryos of various sizes and globular embryos were observed on the surface of the immature zygotic embryos, they later developed to produce heart, torpedo and cotyledonary embryos (Figure 1I). Embryos were transferred to proliferating medium where secondary embryogenesis was observed and after repetitive subcultures the existing embryos could proliferate by producing embryogenic masses (“ms” in Figure 1J) from which new embryos were originated (arrow in Figure 1J).

The cells of early multicellular embryos of this somatic embryogenesis system showed a similar structural organization than those of the microspore embryogenesis pathway, early embryo cells exhibited small size, dense cytoplasm with small vacuoles and a mid-size nucleus (“emb” in Figure 1K, L), clearly distinguishable from the larger cells displayed by the immature zygotic embryo which exhibited a large vacuole (“zye” in Figure 1K). Secondary embryos of microspore and somatic origin emerged and developed from the embryogenic masses as small multicellular embryos (“emb” in Figure 1M) formed by cells which exhibited similar organization than described for cells of early multicellular embryos directly originated from microspores or immature zygotic embryos, very different than the larger and highly vacuolated cells of the embryogenic masses (“ms” in Figure 1M).

### DNA methylation changes during early embryogenesis of both microspore and zygotic embryo origins

**Figure 2:**
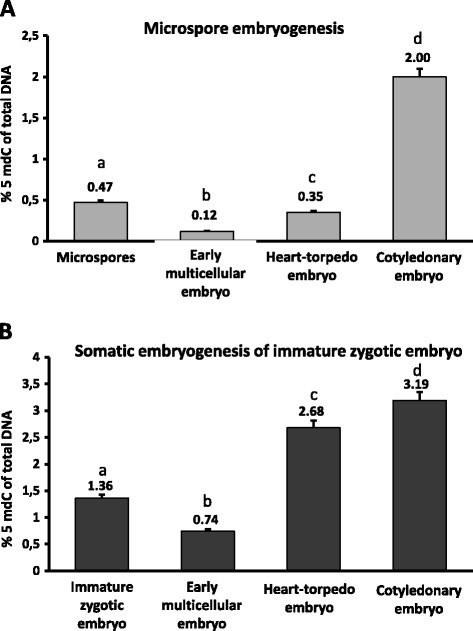
**Global DNA methylation during** ***in vitro*** **embryogenesis of microspore and immature zygotic embryo.** Histograms representing the mean values of 5mdC percentage of total DNA in different stages of microspore embryogenesis **(A)** and somatic embryogenesis of immature zygotic embryos **(B)**, quantified by ELISA-based immunoassay. Bars on columns represent the standard errors of the means. Different letters on columns indicate significant differences according to ANOVA and Tukey’s test at *P ≤* 0.05.

The percentage of global DNA methylation was analyzed at different developmental stages of embryogenesis as well as in microspores and zygotic embryos (Figure 2). The results of the quantification showed similar temporal DNA methylation profiles in both embryogenesis pathways. DNA methylation significantly decreased (*p* ≤ 0.05) after embryogenesis induction, in early multicellular embryos, in comparison with microspores in anthers, and zygotic embryos before embryogenesis initiation. At later embryogenesis stages DNA methylation levels significantly increased (*p* ≤ 0.05) in heart and torpedo embryos, and during late embryo development, reaching the highest level in cotyledonary embryos in the two *in vitro* systems (Figure 2). In heart-torpedo embryos of the second pathway, derived from zygotic embryos, the increase in global DNA methylation was higher than in the microspore embryogenesis pathway.

**Figure 3:**
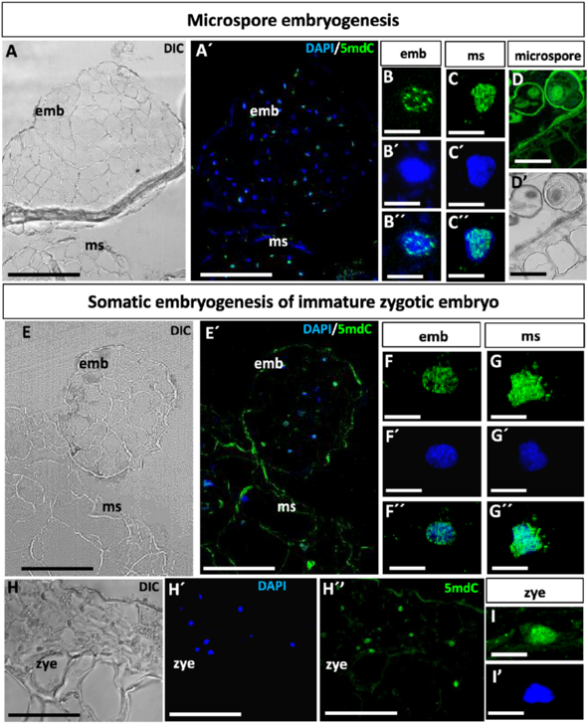
**Methylated DNA nuclear patterns at early** ***in vitro*** **embryogenesis of microspore and immature zygotic embryo. A-D**: Microspore embryogenesis. **E-I**: Somatic embryogenesis of immature zygotic embryos. **A**, **A’**, **E**, **E’**: Confocal images of 5mdC immunofluorescence in early multicellular embryos (emb) and embryogenic masses (ms). Differential interference contrast, DIC, images **(A, E)**, and merged images of 5mdC immunofluorescence (green) and DAPI staining (blue) for the nuclei **(A’, E’)** of the same sections. **B-B**”, **C-C**”, **F-F**”, **G-G**”: High magnification images of representative nuclei of early multicellular embryos, “emb” **(B-B”**, **F-F”),** embryogenic masses, “ms” **(C-C**”, **G-G”)**, microspores **(D, D’)** and immature zygotic embryos, “zye” **(I, I’)** showing 5mdC immunofluorescence in green **(B, C, D,F, G, I)**, DAPI staining of nuclei in blue **(B’**, **C’**, **F’**, **G’**, **I’)** and merged images of both green and blue channels **(B”**, **C”**, **F”**, **G”)**. Bars: **A, A´, E, E**´: 50 μm; **B, B´,B´´**,**C**, **C´**, **C´´**, **F**, **F´**, **F´´**, **G**, **G’**, **G”**, **I**, **I’**: 5 μm; **D**, **D’**: 10 μm.

To analyze the changes in the nuclear distribution pattern of methylated DNA during early embryogenesis, immunofluorescence with anti-5mdC antibody followed by confocal analysis was performed at early stages of both primary and secondary embryogenesis, specifically at the stage of early multicellular embryos in the two pathways studied, microspores and immature zygotic embryos. The 5mdC immunofluorescence signal was similar in early multicellular embryo cells of the two embryogenesis pathways (Figure 3) and in primary and secondary embryos. The 5mdC immunofluorescence signal was very different in early embryo cells than inf microspores of anthers (Figure 3D, D’), immature zygotic embryos (“zye” in Figure 3H, H’, H”, I, I’) and embryogenic masses (“ms” in Figure 3A, A’, C, C’, C”, E, E’, G, G’, G”). Early multicellular embryos directly originated from *in vitro* microspore or zygotic embryo cultures by primary embryogenesis showed the same 5mdC pattern of localization than multicellular embryos (“emb” in Figure 3A, A’, E, E’) produced from embryogenic masses (“ms” in Figure 3A, A’, E, E’) by secondary embryogenesis in the two pathways. Early embryo cells displayed a distribution pattern of 5mdC immunofluorescence in small bright spots over the nucleus, which were clearly identified by DAPI staining (Figure 3, B, B’, B”, , F, F’, F”), these 5mdC fluorescence spots probably correspond to small heterochromatin masses. On the contrary, nuclei of microspores (Figure 3D, D’), immature zygotic embryos (“zye” in Figure 3H, H’, H”, I, I’) and embryogenic masses (Figure 3C, C’, C”, G, G’. G”) showed an intense 5mdC immunofluorescence signal which covered almost the entire nuclear region. The exine, the special pollen wall, showed unspecific autofluorescence (Figure 3D) which did not interfere with the specific immunofluorescence signals detected throughout the microspore embryogenesis analysis. Control experiments avoiding the first antibody or the denaturation step, required for 5mdC recognition by the antibody, did not show labeling in any sample and developmental stage, assessing the specificity of the antibody.

### Changes in pectin esterification of cell walls during early embryogenesis of both microspore and zygotic embryo origins

**Figure 4:**
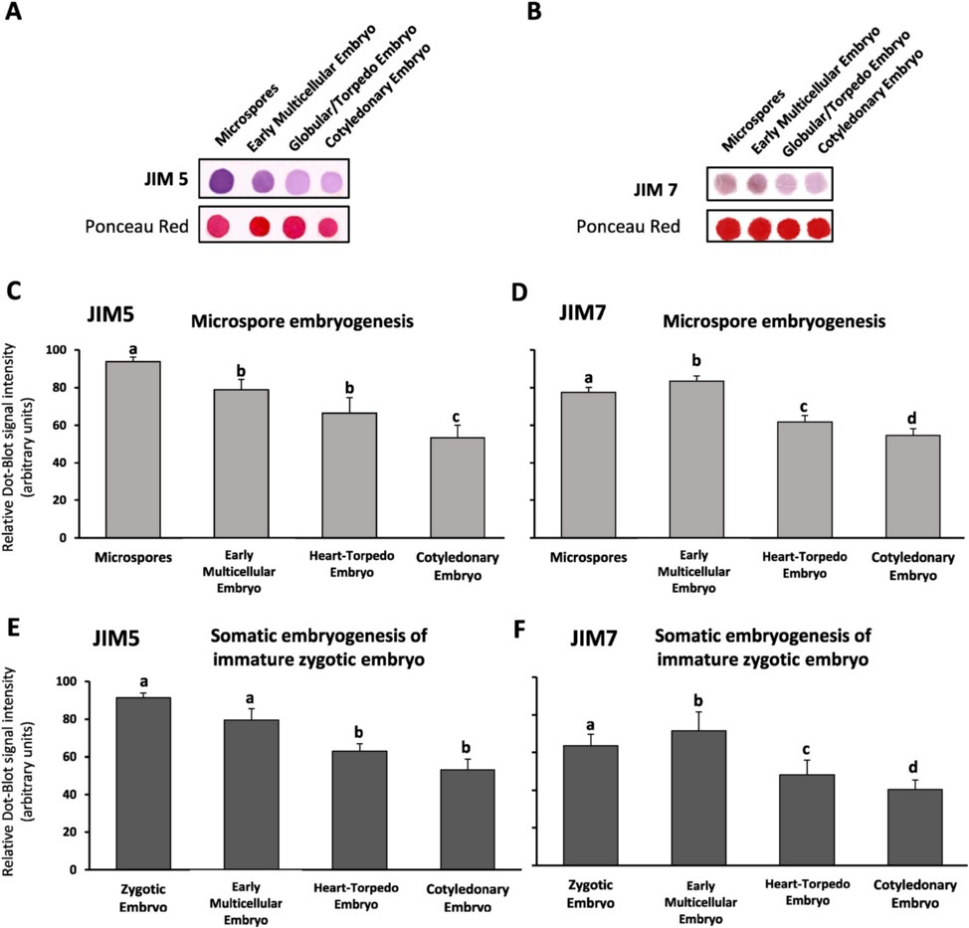
**Distribution patterns of non-esterified (JIM5) and highly-esterified (JIM7) pectins during** ***in vitro*** **embryogenesis of microspore and zygotic embryo origins. A, B**: Representative immuno dot-blot assays of JIM5 **(A)** and JIM7 **(B)** at different developmental stages of microspore embryogenesis. Ponceau Red staining for total proteins and immuno-dot-blot of the same strip is shown for each antibody. **C-F**: Histograms representing the mean values of dot color intensities in arbitrary units of JIM5 **(C, E)** and JIM7 **(D, F)** during microspore embryogenesis **(C, D)** and somatic embryogenesis of immature zygotic embryos **(E, F)**. Bars on columns indicate standard errors of the means. Different letters on columns indicate significant differences according to ANOVA and Tukey’s test at *P* ≤ 0.05.

The changes in the proportion of pectin esterification in the cell walls during early stages of embryogenesis of microspore and zygotic embryos were analyzed by using JIM5 and JIM7 specific antibodies, which recognize non-esterified and highly-esterified pectins respectively, in immunodot-blot and immunofluorescence assays. Dot-blot analysis was performed in microspores and zygotic embryos before embryogenesis induction as well as in embryos during consecutive developmental stages of both embryogenic pathways. The differences in the signal intensities of dot-blots, which were performed over equal amounts of protein extracts, as illustrated by the Ponceau red staining, revealed changes in the proportion of highly-esterified and non-esterified pectins of cell walls during embryogenesis (Figure 4A, B). The quantification of the signal intensities showed similar temporal profiles in the two embryogenesis pathways for each antibody (Figure 4C, D, E, F). JIM5 signal, corresponding to non-esterified pectins, was high in microspores and zygotic embryos before induction whereas it progressively diminished with embryogenesis progression in heart, torpedo and cotyledonary embryos (Figure 4C, E); in the microspore embryogenesis pathway, the lowering of JIM5 signal was significant also at earlier stages, in multicellular embryos (Figure 4C, E). On the contrary, JIM7 antibody which recognizes highly-esterified pectins, showed a significant signal increase (*p* ≤ 0.05) in early multicellular embryos in comparison with microspores and immature zygotic embryos (Figure 4D, F). The intensities of JIM7 signals at advanced embryogenesis stages progressively decreased, in heart, torpedo and cotyledonary embryos (Figure 4D, F). Control experiments avoiding the first antibodies did not show labeling in any case.

**Figure 5:**
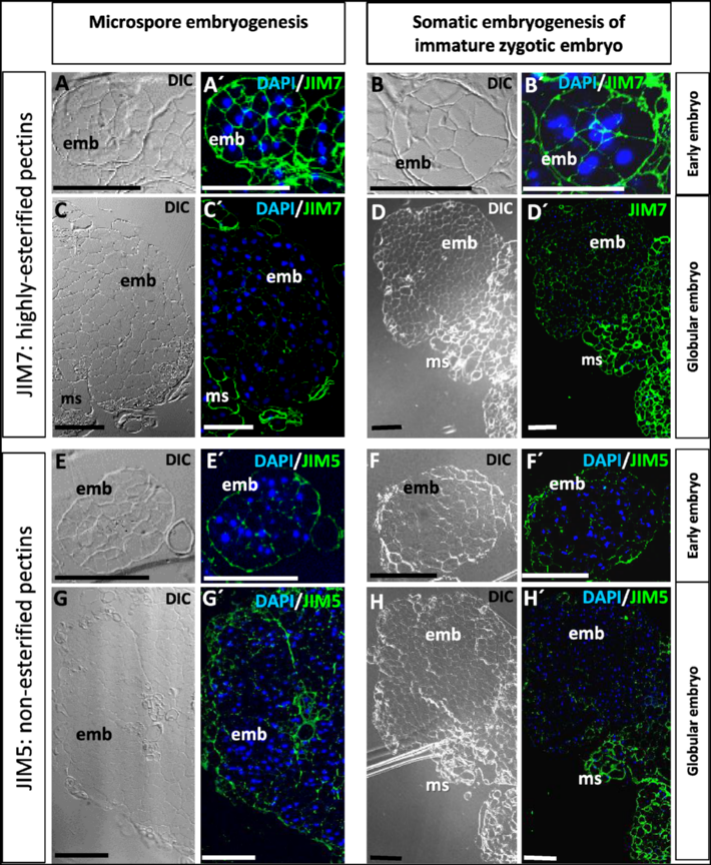
**Immunolocalization of non-esterified (JIM5) and highly-esterified (JIM7) pectins at early** ***in vitro*** **embryogenesis of microspore and immature zygotic embryos. A, A’, C, C’, E, E’, G, G’**: Microspore embryogenesis. **B, B’, D, D’, F, F’, H, H’**: Somatic embryogenesis of immature zygotic embryos. **A’-H’**: Confocal merged images of immunofluorescence signal (green) of JIM7 (**A’-D’**) or JIM5 (**E’-H**’) combined with DAPI staining of nuclei (blue). **A-H:** Differential interference contrast, DIC, images of the same sections. **A, A’, B, B’, E, E’, F, F**’: Early multicellular embryos. **C, C’, D, D’, G, G’, H, H’**: Large multicellular and globular embryos (emb) emerging from embryogenic masses (ms) by secondary embryogenesis. Bars: 100 μm.

Immunofluorescence assays revealed analogous localization patterns in both embryogenesis pathways from microspore and immature zygotic embryos for each pectin epitope, non-esterified (JIM5 labelling) and highly-esterified (JIM7 labelling) pectins. JIM7 labelling was intense on peripheral and inner cell walls of early multicellular embryos (“emb” in Figure 5A, A’, B, B’). In contrast, early multicellular embryos showed very low or no labeling with JIM5 antibody on inner cell walls, only the peripheral wall layer surrounding the multicellular embryos exhibited JIM5 labelling (Figure 5E, E’, F, F’). At more advanced developmental stages, globular embryos developed by direct and secondary embryogenesis showed very low or no labeling with JIM7 or JIM5 antibodies, whereas cell walls of embryogenic masses appeared labeled with both antibodies (“ms” in Figure 5C, C’, D, D’, H, H’). The fact that the results of the dot-blot assays showed faint positive JIM5 and JIM7 signals on advanced embryo stages indicated a higher sensitivity of this assay in comparison with immunofluorescence on resin sections where only epitopes exposed on the section surface can be detected; on the other hand, the possibility that some cells of the embryogenic masses would remain attached to embryos in the samples used for dot-blot assays and slightly contribute to dot-blot signals, cannot be completely excluded.

### Localization of endogenous auxin during early embryogenesis of both microspore and zygotic embryo origins

**Figure 6:**
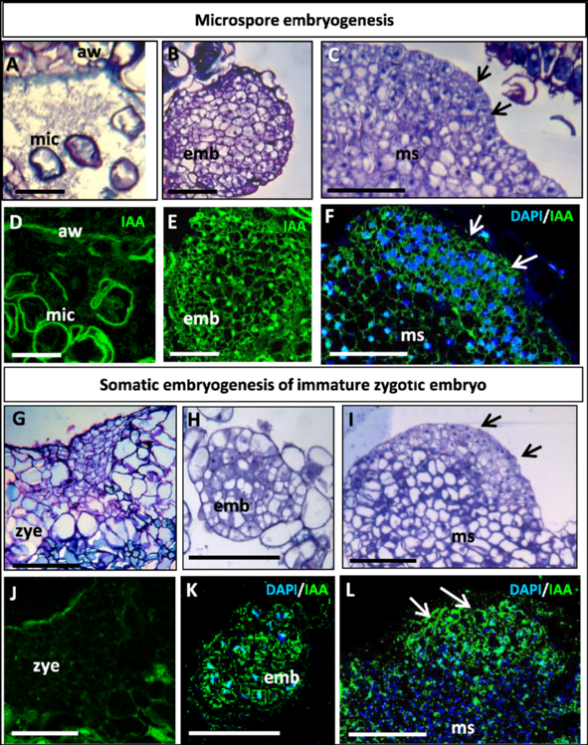
**Immunolocalization of auxin (IAA) at early** ***in vitro*** **embryogenesis of microspore and immature zygotic embryos. A-F**: Microspore embryogenesis. **G-L**: Somatic embryogenesis of immature zygotic embryos. **A, B, C, G, H, I**: Resin sections stained by toluidine blue. **D, E, F, J, K, L**: Confocal images of IAA immunofluorescence signal (green); in some images, DAPI staining of nuclei (blue) is also shown merged with the green IAA signal (**F, K, L**).**A, D**: Microspores (mic). **B, E, H, K**: Early multicellular embryos (emb). **C, F, I, L**: Dense cells (arrows) from which early embryos originate at the surface of embryogenic masses (ms). Bars **A, D**: 50 μm; **B, C, E-L**: 100 μm.

The localization of the endogenous auxin indol acetic acid, IAA, was approached by immunofluorescence assays using specific anti-IAA antibodies. Results showed similar patterns of IAA localization in the two embryogenesis pathways, from microspores and from zygotic embryos. After embryogenesis induction, very low or no IAA labeling was observed in microspores (Figure 6A, D) and immature zygotic embryos (“zye” in Figure 6G, J) in culture. In microspores, the special pollen wall, the exine, showed unspecific autofluorescence (Figure 6D). However, an intense IAA labeling was found in the cytoplasm of cells of early multicellular embryos produced in the two pathways by primary embryogenesis (“emb” in Figure 6B, E, H, K) B, F). During early secondary embryogenesis, at the surface of the embryogenic masses (“ms” in Figure 6C, F, I, L) the dense cells which originate new multicellular embryos, also showed intense IAA labeling (arrows in Figure 6C, F, I, L), whereas the subjacent embryogenic mass cells (“ms” in Figure 6C, F, I, L) did not show any labeling. Controls by immunodepletion of the IAA antibody did not show labeling in any cellular structure and developmental stage, supporting the specificity of the immunofluorescence results.

**Figure 7:**
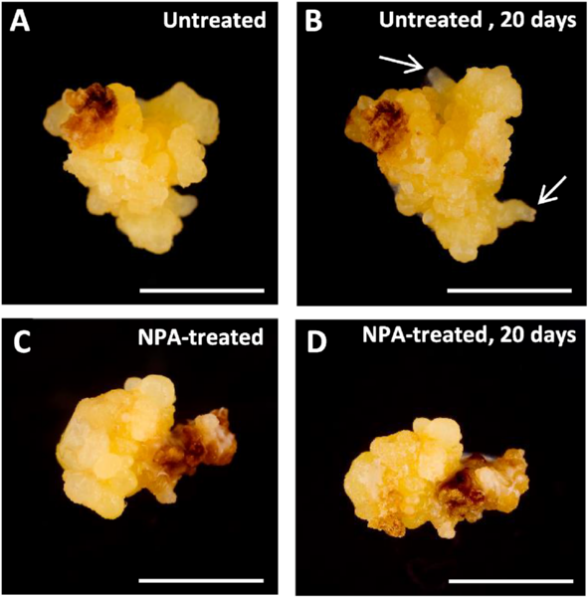
**Effects of inhibiting auxin transport by NPA on** ***in vitro*** **embryogenesis initiation and progression.** Embryogenic masses originated by secondary embryogenesis from immature zygotic embryos. **A, B**: Control cultures, without NPA, at the beginning of the treatment **(A)** and 20 days afterwards **(B)**, when embryo development and new embryos (arrows) were observed. **C, D**: Culture containing 40 μM NPA at the beginning of the treatment **(C)** and 20 days afterwards **(D)**, no further growth or new embryos were observed. Bars 5 mm.

To assess the involvement of endogenous auxin accumulations in the early stages of embryogenesis of the two pathways, treatments with the inhibitor of the polar auxin transport 1-N-naphthylphthalamic acid (NPA) were performed to *in vitro* cultures with embryogenic masses originated from the two systems. Results were similar in embryogenic cultures of both origins microspore and immature zygotic embryos; they showed that NPA treatment affected embryo development in comparison with control cultures (Figure 7). After 20 days of NPA treatment, neither new embryos nor development of the embryogenic masses were observed on treated cultures, whereas in parallel embryogenic cultures without NPA embryogenesis normally initiated and progressed. New embryos emerging from the embryogenic masses and development of previous embryos were observed at the same time period (Figure 7).

### Distribution patterns of the cellular markers used (methylated DNA, highly-esterified and non-esterified pectins, and auxin) in other developmental systems with proliferating and differentiated cells

In order to evaluate the patterns of the selected cellular features (5mdC, highly-esterified and non-esterified pectins, and IAA) as early markers of embryogenesis, their distribution was analyzed in other two plant developmental systems with proliferating and differentiated cells, taken as positive and negative controls. One system was the proliferating cells of developing zygotic embryos in which the markers should be found, and in a different plant species, *Brassica napus*, to additionally evaluate whether those features were also present in other species. The other system was a non-embryogenic system with differentiated cells, the epidermis and subjacent cell layers of mature anthers, differentiated tissues where the markers should not be present.

**Figure 8:**
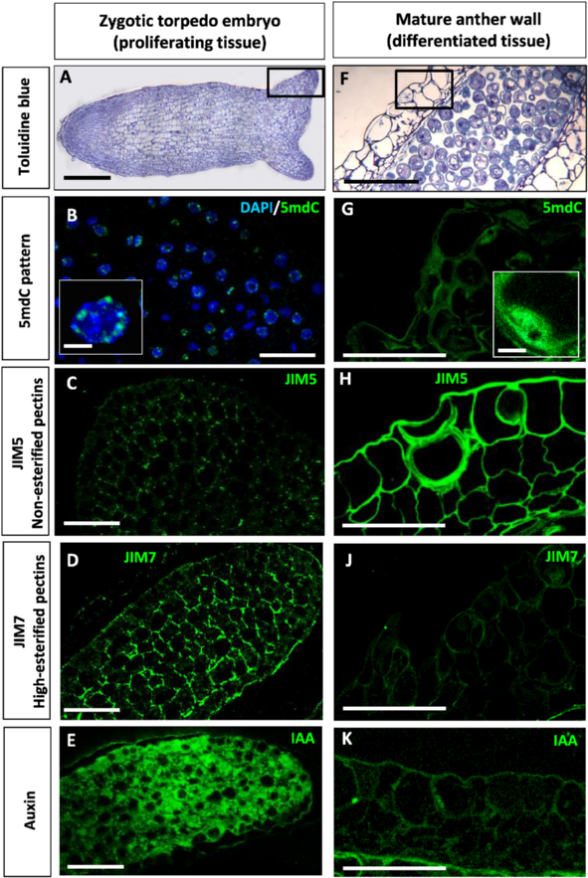
**Immunolocalization of 5mdC, esterified (JIM7) and non-esterified (JIM5) pectins, and IAA in other systems. A-E**: Zygotic torpedo embryo of *Brassica napus.* **F-K**: Mature anther of *Quercus suber.* **A, F**: Panoramic views of zygotic torpedo embryo and mature anther, resin sections stained by toluidine blue; squares in **A** and **F** indicate representative regions of proliferating embryo cells (A) and differentiated cells of anther wall (B) where the analysis is focused. **B-E, G-K**: Higher magnifications of immunofluorescence signals (green) for 5mdC (B, G), JIM5 (C, H), JIM7 (D, J) and IAA (E, K) antibodies. In **B**, DAPI staining for nuclei (blue) is merged with 5mdC immunofluorescence (green). Insets in **B** and **G** show representative nuclei of each cell types, at high magnification. Bars: **A, F**: 50 μm, **B-E, G-K**: 25 μm, insets: 5 μm.

The results of the comparative analysis between the two systems, proliferating embryo cells and non-embryogenic differentiated cells are shown in Figure 8. In proliferating cells of the zygotic torpedo embryos (Figure 8A), the patterns of immunolocalization observed with all antibodies were similar than the patterns found in early multicellular embryos developed *in vitro*. Methylated DNA, localized by anti-5mdC immunofluorescence, was scarce and distributed in small spots over nuclei (Figure 8B). JIM5 and JIM7 antibodies revealing non-esterified and highly-esterified pectins respectively, exhibited localization patterns in which the walls of proliferating embryo cells appeared higher labeled with JIM7 than with JIM5 (Figure 8C, D) indicating a higher proportion of esterified pectins in these cells. Auxine immunofluorescence signal was intense in the cytoplasm of the proliferating embryo cells (Figure 8E) localized at the developing cotyledon tips of the torpedo embryo. On the contrary, the patterns of localization in the differentiated tissue of the anther wall (Figure 8F) were opposite to those found in embryo cells. 5mdC signal was intense and covered the whole nuclear area (Figure 8G), corresponding to abundant hypermethylated DNA. The cell walls of the anther tissues showed intense JIM5 signal and very low JIM7 signal which indicated a higher proportion of non-esterified pectins in these cell walls. Very low or no IAA signal was observed in differentiated cells of the anther wall layers (Figure 8J).

## Discussion

### Global DNA methylation decrease accompanies nuclear remodeling in early embryo cells

DNA methylation plays an essential role in plant growth as a mechanism to epigenetically maintain developmental fates in proliferating and differentiating cells [14,15,32-34]. In the present work, DNA methylation level and distribution pattern, in relation to chromatin changes, have been analyzed during early embryogenesis of the two pathways, of microspores and zygotic embryos, results suggesting that the initiation of both embryogenic processes is epigenetically regulated. Differences in global DNA methylation levels and changes in the 5-methyl-deoxy-cytidine (5mdC) distribution patterns were found in the early embryo cells of the two *in vitro* embryogenesis pathways analyzed, suggesting changes of nuclear activity. A significant decrease in global DNA methylation was observed in early multicellular embryos in comparison with microspores, immature zygotic embryos or embryogenic mass cells from both embryogenesis *in vitro* systems. Reports in tree species showed that the morphogenic capacity was associated with low levels of DNA methylation [32] and a transient DNA demethylation of the ovules occurred after pollination [35]. In *Brassica napus*, a decrease of DNA methylation has been also reported at early stages of microspore embryogenesis [14].

The immunolocalization essays showed not only a decrease on the 5mdC labelling in the cells of early embryos derived from microspores and zygotic embryos, but also a change in the distribution pattern of 5mdC in small nuclear spots, in contrast with the 5mdC distribution pattern over large nuclear regions corresponding with highly condensed chromatin masses of non-embryo cells. This 5mdC distribution pattern over small heterochromatin masses correlates with the pattern found in active proliferating cells of root meristems [34], and early microspore-derived embryos of rapeseed, *Brassica napus*, with high transcriptional activity and decondensed chromatin patterns [11,14]. In contrast, 5mdC distribution over large heterochromatin masses has been associated with decreased cellular activity stages and cell differentiation, as in the generative/sperm cells of pollen grains, in the differentiating cells of advanced embryos [14,15] or in the tapetal cells during the high chromatin condensation occurring during their developmental programmed cell death [36].

The data presented here revealed an epigenetic change associated with the switch towards the embryogenic developmental program and with the activation of cell proliferation occurring at the beginning of embryogenesis in the two pathways studied, microspore embryogenesis through anther culture and somatic embryogenesis from immature zygotic embryos, for the first time in a woody species, cork oak.

### Cell walls of early embryo cells exhibit higher levels of esterified pectins

Increasing evidence has linked the cell wall remodeling with many processes involved in plant growth and development, including somatic embryogenesis [22,37,38]. Several monoclonal antibodies have been used to unravel the involvement of specific cell wall epitopes in controlling cell growth and morphogenesis; specifically, there are different studies on antigen distribution detected by the antibodies JIM5 and JIM7 recognizing non-esterified and highly-esterified pectins respectively, in several plant tissues and organs [20,21,39]. In the present work, we have used both JIM5 and JIM7 antibodies to analyze changes in cell wall associated with *in vitro* embryogenesis initiation in *Quercus suber* L.

In very young microspore-derived embryos of herbaceous species (*Capsicum annuum* and *Brassica napus*), a high level of esterified pectins was observed in the cell walls [17,18,40]. Using an *in situ* localization approach at both light and electron microscopy levels, it has been reported that in walls of proliferating cells, the levels of esterified pectins were higher than in the differentiating cells, which showed cell walls rich in non-esterified pectins [17,18]. Differences in the proportion of esterified pectins between microspores and microspore-derived embryos were reported in tree species, such as *Quercus suber*, *Citrus clementina*, and *Olea europaea* [8,10,23].

The results presented here showed differences in the distribution pattern of highly-esterified pectins in early embryo cells in comparison with non-embryogenic cells, in the two pathways (embryogenesis of microspores and zygotic embryos) in cork oak. Highly-esterified pectins were more abundant in early embryo cell walls than in microspores, zygotic embryo and embryogenic mass cells, while they diminished with embryo development and differentiation. This evidence was consistent with the abundance of esterified pectins in the newly-formed walls of proliferating cells of young microspore-derived embryos and root tip meristematic cells of some herbaceous species [17,18], thus providing experimental support indicating that a high proportion of esterified pectins in walls is not only a marker for proliferation events, but also an early marker of both reprogramming pathways to embryogenesis, of microspores and immature zygotic embryos origins.

Our results showed that, in contrast with the inner cell walls, the peripheral wall layer that surrounds the early multicellular embryos was labeled by JIM5. Previous reports have described the presence of a thick wall underneath the exine, the special microspore wall, in embryogenic microspores and young embryos of various species, like *Capsicum annuum* and *Olea europaea* [9,10]. The development of those peripheral thick wall in early stages of somatic embryogenesis and organogenesis, and also microspore embryogenesis, has been reported as a specific feature of these processes [9,10,16], though the nature of this wall has not been elucidated yet. It has been proposed that this layer could constitute an organized network with differential wall components which may create a specific cellular environment with altered permeability and receptors, providing conditions to express morphogenic competence [16]. The present study reveals that at early stages of the two *Quercus suber in vitro* embryogenesis pathways, from microspores and immature zygotic embryos, there is also a peripheral wall in early multicellular embryos with a differentially higher proportion of non-esterified pectins than the inner cell walls, providing additional evidences of the existence of special wall structures surrounding groups of cells committed for entering the cell cycle and initiate the embryogenic program.

### High endogenous auxin level is present in early embryo cells

Many aspects of auxin action depend on its local biosynthesis and differential distribution within plant tissues, mainly regulated by its directional transport between cells [41]. We have analyzed the endogenous auxin IAA distribution and level during early stages in the two embryogenesis pathways, from microspore and immature zigotic embryos, in cork oak, *in vitro* systems in which the embryo development occurs in a medium free of exogenous IAA [42,43]. Interestingly, the IAA immunolocalization assays revealed a differential and significant increase of the IAA endogenous levels in the early multicellular embryo cells, in comparison with the microspores, immature zygotic embryos and embryogenic mass cells which showed very low IAA signal. In *Arabidopsis*, auxin has been localized by the expression of the auxin-responsive promoter DR5 in cells of the embryo proper at very early stages [24,44]. IAA biosynthesis is associated with rapidly dividing and rapidly growing tissues, especially in shoots and roots, free auxin concentrations in the living plant are in the apical meristems of shoots, young leaves, root meristems and lateral root initiation sites [45,46]. In a new *Brassica napus* microspore embryogenesis *in vitro* system at low temperature, our group reported preliminary results showing that IAA also localized in cells of early multicellular embryos [28], similarly to early zygotic embryogenesis.

The results presented here indicate the increase of endogenous levels of IAA in the formation of early multicellular embryos of both microspore and zygotic embryo origins, in *Quercus suber*. This result, together with the fact that the inhibition of auxin transport by NPA negatively affected embryogenesis initiation and development suggest that endogenous IAA biosynthesis and transport would be involved in the activation of proliferation and the switch of the embryogenic program of the reprogrammed microspores, zygotic embryo and embryogenic mass cells.

### Early embryogenesis from microspores and zygotic embryos exhibits similar early markers: epigenetic changes by DNA hypomethylation, cell wall remodelling by high pectin esterification and high endogenous auxin levels

*In vitro* embryogenesis initiation and progression is conditioned by many factors, still largely unknown. The comparative analysis performed here during the early stages of embryogenesis in two different *in vitro* systems, microspore embryogenesis through anther cultures and somatic embryogenesis from immature zygotic embryo cultures, in a woody species, *Quercus suber*, has permitted the identification of differential cell features, summarized in Table 1, specific to the reprogramming process towards embryogenesis, as early markers which are similar in both embryogenesis pathways from microspore and immature zygotic embryos in cork oak. These differential features are the decrease of DNA methylation, the remodeling of cell walls by increasing the proportion of esterified pectins, and the accumulation of endogenous auxin in the cells that initiate the embryogenic program. The present work provides new evidences of two important questions for the understanding of the *in vitro* embryogenesis process. On one hand, the reported data reveal that several basic cellular features undergo similar changes in the two different embryogenesis pathways studied at early stages, indicating that basic cellular processes underlying early *in vitro* embryogenesis are common to various systems and suggesting that general mechanisms could regulate the process independently of the cell type origin and culture conditions . Moreover, the results illustrate the relevance of three cellular processes which specifically occur in early embryo cells just after induction, while the same cellular changes are not observed neither before embryogenesis initiation neither at advanced developmental stages, fact that permits to consider these features (DNA hypomethylation, cell wall remodeling by high esterification of pectins, and endogenous auxin increase) as early markers of the process, opening the possibility to design strategies for efficient manipulation of *in vitro* protocols to improve yields.

**Table 1:** **Early markers present in** ***in vitro*** **embryogenesis of both microspores and immature zygotic embryos origins**

**Early markers**	**Before embryogenesis induction**	**After embryogenesis induction**
	**Cells from which embryos originate (microspores, immature zygotic embryos, embryogenic masses)**	**Early embryos (derived from microspores, immature zygotic embryos and embryogenic masses)**
DNA methylation level	High (++)	Low (+/−)
DNA methylation pattern	Large chromatin masses	Small chromatin masses
Highly-esterified pectins in cell walls	Mid (+)	High (++)
Peripheral wall layer (rich in non-esterified pectins)	Not present (−)	Present (+)
Endogenous auxin	Low (+/−)	High (++)

A previous cytochemical and immunocytochemical study compared the structural organization of advanced globular embryos of both origins, microspores and immature zygotic embryos, finding similar anatomy and cellular organization patterns in both of them; the common features found concerned high vacuolization, small nuclear size and structure, abundant ribosomal population, and presence of starch granules [7]. Studies in stress-induced microspore embryogenesis and somatic embryogenesis of other species have indicated the involvement of epigenetic marks [14,15], cell wall components [10,16-19] and endogenous phytohormones [27,28,47] in the initiation and progression of organogenesis and embryogenesis. Some of the markers described here in cork oak have been also found in other plant species, for example, DNA hypomethylation has been observed in microspore embryos of *Brassica napus* [14] and *Hordeum vulgare* [15], a high proportion of esterified pectins in cell walls was described in microspore embryos of *Capsicum annum* [17,18], and production of endogenous auxin in early embryos was found in microspore embryogenesis of rapeseed [28]. T he present work is the first report on microspore and somatic embryogenesis in a forest tree, that shows all these cellular processes during the initiation of two different embryogenesis pathways which are induced by different *in vitro* protocols. Interestingly, the reported cell modifications were observed not only in early embryos originated directly from microspore and zygotic embryos, but also in secondary embryos produced from embryogenic masses, at early stages. In contrast, these features were not found in the cells that originated the embryos (microspores and immature zygotic embryo cells) before embryogenesis induction. Moreover, they were also found in proliferating cells of zygotic embryos of other plant species, *B. napus*, whereas differentiated cells of mature organs, as revealed in mature anthers here, showed opposite features than early embryo cells regarding DNA methylation, pectin esterification and endogenous auxin content. The fact that the same differential cell features appear in the two early embryo cells of both origins, from microspores and immature zygotic embryos, produced by primary or secondary embryogenesis indicate that they can be considered as early markers of *in vitro* embryogenesis. As stated before, some of them have been reported individually in embryos of other plants [14,15,17,18,28] and, in the present work, the three features were found on proliferating cells of zygotic embryos in a different species, *Brassica napus*, these data support the idea that these early markers reflect a general behavior that could be exploited for other species.

Increasing evidences have documented the effect of different phytohormones, including auxin, in signaling processes of cell wall remodeling, necessary for cell elongation and growth, and in chromatin remodeling for the activation of specific gene expression developmental programs. The changes reported here affecting epigenetic marks and cell wall polymers are associated with the accumulation of auxin in early embryo cells, this data could reflect different processes that are interconnected in complex regulatory mechanisms that control cell reprogramming and embryogenesis initiation.

## Conclusions

The identification of early markers of embryogenesis can help to monitor the metabolic processes involved in the induction providing a wider understanding of the physiology and mechanisms controlling plant cell reprogramming and embryogenic competence acquisition. The present work is the first report that shows, by a comparative analysis during the early stages of embryogenesis, that various cellular processes, DNA hypomethylation, cell wall remodeling by increasing pectin esterification, and endogenous auxin increase, occur concomitantly at early stages of two embryogenesis pathways, microspore embryogenesis and embryogenesis from immature zygotic embryos, which are induced by different *in vitro* protocols, identifying similar early embryogenic markers in *Quercus suber*. The data reported here, in a woody species, which have shown to be extremely recalcitrant to *in vitro* embryogenesis, is of particular relevance. The information gained will be useful to increase our knowledge on the regulatory mechanisms and involved factors in the *in vitro* induction of embryogenesis, opening the door to identify novel strategies and selective targets for improving the efficiency of the process in biotechnology, agronomic and forestry breeding programs.

## Methods

### Plant material

Catkins and immature pollinated acorns were collected from *Quercus suber* L. trees every week during flowering period and fruit development period respectively from two selected trees in the E.T.S.I. de Montes (Universidad Politécnica de Madrid) and three selected trees from El Pardo, Madrid, Spain.

The branches bearing catkins were pre-treated by chilling in darkness with moist cotton at 4°C for 1 week. Catkins of different sizes were squashed and stained with DAPI (4, 6-diamidino-2-phenylindole) to visualize the nuclei and observed under fluorescence microscope to determine the developmental stage of the microspore. The selected catkins, only those red coloured and 1.5-2.5 cm long that contains the vacuolated microspore, the most responsive stage for embryogenesis induction [48], were surface sterilized. Immature acorns selected at the appropriate developmental stage for somatic embryogenesis induction were those with small size, 1 cm, and green colour; they were kept at 4°C for one week. Catkins and acorns were surface sterilized by immersion in 70% ethanol (Merck), for 1 min, and 2% sodium hypochlorite with a drop of Tween-20 for 20 min followed by three rinses in sterile distilled water for 10 min each. The anthers and the immature zygotic embryos were carefully excised from catkins and acorns respectively, isolated, dissected and cultured under aseptic conditions in a laminar flow cabinet.

For their use as controls in other plant systems, some mature anthers of *Quercus suber* were extracted directly from catkins, without any pretreatment, and fixed and processed for further use in immunofluorescence assays. Also, zygotic embryos at the torpedo stage were extracted from siliques of *Brassica napus* plants, fixed and processed for immunofluorescence.

### Culture media

Basal culture medium for both microspore and somatic embryogenesis contained full macronutrients [49], micronutrients and cofactors [50], 3% sucrose and was solidified with 0.8% Agar, adjusted to pH = 5.6, and autoclaved. The amino acids and labile compounds were added after autoclaving by filter-sterilization (0.22 μm). Microspore embryogenesis induction medium was basal culture medium supplemented with 1% active charcoal, while somatic embryogenesis induction medium was supplemented with 0.5 mg/L 2,4-D.

### *In vitro* embryogenesis of microspores and immature zygotic embryos

Anthers containing vacuolated microspores were isolated from the catkins and plated in Petri dishes with induction medium for microspore embryogenesis in an amount of 100 anthers per plate approximately, subjected at 32°C in darkness during 5 days [43], and then transferred to 25°C. After one month visible embryos emerged from inside the anther. Embryos obtained were isolated and transferred to plates containing basal medium without active charcoal and supplemented with 0.5 g/L glutamine, where the embryos were propagated by secondary embryogenesis.

Immature zygotic embryos were cultured in Petri dishes containing induction media for somatic embryogenesis placing 5 embryos per plate and cultured at 25°C with 16/8 h light/darkness, for one month [42,51]. Then, the immature zygotic embryos were transferred to growth regulator-free medium, where new embryos and embryogenic masses, were found and suffered recurrent somatic embryogenesis process.

Every week, embryogenesis cultures, were observed under a microscope Leica MZ16FA, to monitor the embryogenesis process. Culture samples at different developmental stages of the two embryogenic systems were either fixed for microscopic analysis or frozen by immersion in liquid nitrogen for biochemical assays.

### Treatments with NPA

*N*-1-Naphthylphthalamidic acid, NPA (Duchefa), auxin transport inhibitor, was added at 40 μM concentration to the corresponding media of microspore and somatic embryogenesis, using a stock of 0.1 M NPA in DMSO, after filtering with a sterile Ministart filter (Sartorius Biotech). The NPA was added to plates of propagating embryogenic masses by secondary embryogenesis, using parallel plates of the same cultures and keeping some plates lacking NPA as controls. To asses NPA effects on embryogenesis development, the embryo growth and emergency of new embryos per plate was evaluated after 20 days in NPA-treated and control cultures.

### Antibodies

The following antibodies and dilutions were used: anti-5-methyl-deoxy-cytidine (5mdC) mouse monoclonal diluted 1:100 (Eurogentec, Cat. N: BI-MECY-0100, Liege, Belgium), anti-IAA mouse monoclonal diluted 1:100 (Sigma, Cat. N: A 0855), JIM5 rat monoclonal (Plant Probes, Cat. N: JIM5), and JIM7 rat monoclonal (Plant Probes, Cat N: JIM7) diluted 1:25 for immunofluorescence, and 1:100 for immunodot-blot assays.

### Fixation and processing for immunofluorescence

Embryogenesis culture samples from microspores and immature zygotic embryos were collected at different times and fixed overnight with 4% paraformaldehyde in phosphate buffered saline (PBS) at 4°C. For control experiments, mature anthers of *Quercus suber* and zygotic torpedo embryos of *Brassica napus* were excised from plants and fixed by the same procedure. Most samples were embedded in resins and some large samples were processed for cryostat.

#### Processing for cryostat sectioning

Fixed samples were washed in PBS, cryoprotected through a gradual infiltration in sucrose solutions: 0.1 M, 0.5 M, 1 M, 1.5 M and 2 M for 1 h each and 2.3 M overnight, at 4°C, embedded in Tissue-Tek optimal cutting temperature (OCT) compound and frozen on dry ice for sectioning in the cryostat (Leica CM 1950). 20–30 μm thick sections were collected on glass slides, washed with water to eliminate the OCT and transferred to a water drop over 3-aminopropyl-triethoxy-silane (APTES)-coated slides, air-dried and stored at −20°C until use for immunofluorescence (IF).

#### Processing for resin embedding and ultramicrotome sectioning

Fixed samples were washed in PBS, dehydrated through an acetone series (30%, 50%, 70%, 90% and 100%) and embedded in Technovit 8100 resin (Kulzer, Germany) at 4°C. The blocks were sectioned at 1–2 μm thickness and stained with 1% toluidine blue for structural analysis, mounted with Eukitt and observed under bright field microscopy. Some sections were placed on APTES coated slides, air-dried, and stored at 4°C until use for immunofluorescence.

### Immunofluorescence

Immunofluorescence (IF) was performed essentially as previously described by us [14,19,28]. For cryostat sections, permeabilization was required prior to IF. After thawing the sections at room temperature, they were dehydrated and rehydrated in a methanol series (30%, 50%, 70%, 90%, 100%, 90%, 70%, 50%, 30%, 5 min each) and PBS. They were subsequently subjected to enzymatic digestion of cell walls for additional permeabilization by treatment with an enzymatic mixture (2.5% pectinase, 2.5% cellulase and 2.5% pectoliase) in PBS for 45 min. Semithin resin sections did not required permeabilization and were subjected directly to the immunodetection, after incubation in PBS for a few minutes. At this step, all section types followed the same protocol of IF. Sections were first blocked with 5% Bovine Serum Albumin (BSA) or 10% foetal calf serum (FCS) for IAA-immunofluorescence, in PBS for 10 min, and incubated for 1 h with JIM5, JIM7, anti-5-methyl-deoxy-cytidine (5mdC), anti-IAA, rat and mouse monoclonal antibodies respectively. For the 5mdC antibody the sections were previously denaturated with 2 N HCl for 45 min. After three rinsing steps in PBS, sections were incubated for 45 min with Alexa Fluor 488-labelled anti-rat or anti-mouse IgG antibody diluted 1:25 in BSA 1% in the dark. After washing in PBS, nuclei were stained with DAPI, washed with PBS, mounted in Mowiol and examined in a confocal microscope (Leica TCS-SP2-AOBS, Vienna, Austria). Optical sections and maximum projections images were obtained with software running in conjunction with the confocal microscope (Leica software LCS version 2.5).

Negative controls were performed as follows: for the 5mdC antibody by avoiding the denaturation step; and for the IAA antibody by immunodeplection assays; the anti-IAA antibody was incubated with a solution of 5 mg/ml synthetic IAA (1:4, v/v) at 4°C overnight. The pre-blocked antibody solution as used as primary antibodies for immunofluorescence, following the same protocol and conditions described above.

### Immuno dot-blot assay for JIM5 and JIM7 antibodies

Samples from the two embryogenesis cultures were collected at different developmental stages, and frozen in liquid nitrogen. Dot-blot assays for JIM5 and JIM7 antibodies were performed as described by us [19]. Samples, 0.1 g for each sample, were homogenized in liquid nitrogen using a chilled mortar and pestle, with 50 ml of buffer containing 50 mM Tris–HCl pH 7.2, 50 mM trans-1,2-diaminocyclohexane-N,N,-tetra-acetic acid (CDTA) and 25 mM dithiothreitol. The resulting supernatant concentrations were determined according to Bradford (Bio-Rad Protein Assay reagent) using bovine serum albumin (BSA) as calibrator and all samples were adjusted to a concentration of 0.5 mg/ml. For immuno Dot-Blot assays, 5 μl aliquots of adjusted supernatants were applied to a nitrocellulose membrane (Millipore; Bedford, MA, United Kingdom) and left to dry for 1 h. Strips were first stained for total protein detection with Ponceau red, and the images of the stained dots were captured by scanning. The membrane was incubated overnight at room temperature, with the primary antibodies (JIM5 and JIM7) diluted 1:100, in the blocking buffer (2% powdered skimmed milk containing 0.05% Tween-20 in PBS), washed, and incubated for 1 h with alkaline phosphatase-conjugated anti-rat antibody diluted 1:1.000 in the blocking solution. Finally, the epitopes recognized by the antibodies were revealed by treatment with a nitroblue tetrazolium, bromo-chloroind-olyl–phosphate (NBT–BCIP) mixture. Quantification of the relative intensity of the dot blot signals was performed by using the tool for color intensity quantification of the image analysis software Adobe Photoshop. For the quantification, images of three replicates for each antibody, developmental stage and embryogenesis pathway were used. Values of color intensity, in arbitrary units, for each antibody were calculated and adjusted to the intensity of Ponceau S dots, which estimate the protein level. Mean values and standard deviations were calculated and the results showed in histograms. Differences among stages were tested by one-way ANOVA analysis of variance followed by Tukey’s multiple comparison test at *P ≤* 0.05.

### Quantification of DNA methylation by 5mdC ELISA-based immunoassay

Samples were collected from both embryogenesis culture systems, from microspores and immature zygotic embryos, at different developmental stages and frozen in liquid nitrogen. Quantification of DNA methylation was performed as previously described [34]. Total DNA was extracted of each sample from 0.1 g of frozen material, using a plant genomic DNA extraction kit (DNeasy Plant Mini, Qiagen) following the kit´s instructions. The purified DNA, 100 ng of genomic DNA for each sample, was used for global DNA methylation quantification by using a MethylFlash Methylated DNA Quantification Kit (Colorimetric, Epigentek, NY) added to an ELISA plate where the methylated fraction of DNA was quantified using 5-deoxy-methyl-cytidine specific antibodies. The amount of methylated DNA was proportional to the optical density (OD) measured in an ELISA plate reader at 450 nm. By subtracting negative control readings from the readings for the sample and the standard, the value of 5-methyl-deoxy-cytosine for each sample was calculated as a ratio of sample OD relative to the standard OD. Results were expressed as the percentage of methylated deoxy-methyl-cytosines (5mdC) of total DNA. For the quantification, three replicates for each developmental stage and embryogenesis pathway were used. Mean values and standard deviations were calculated and the results showed in histograms. Differences among stages were tested by one-way ANOVA analysis of variance followed by Tukey’s multiple comparison test at *P* ≤ 0.05.
